# Etiologies and Risk Factors of Anterior and Posterior Circulation Strokes: A Comparison Study From South India

**DOI:** 10.7759/cureus.66172

**Published:** 2024-08-05

**Authors:** Jeffrin John Varghese, Reji Thomas, S Vijayalekshmi, Sheetal Sasikumar, Jeethu T J, Jisa Merin J N

**Affiliations:** 1 Neurology, Pushpagiri Institute of Medical Sciences & Research Centre, Thiruvalla, IND; 2 Neurology, Tiruvalla Medical Mission Hospital, Thiruvalla, IND

**Keywords:** posterior circulation infarction, anterior circulation infarction, stroke, risk factors, etiology

## Abstract

Introduction: While anterior and posterior circulation strokes share most pathophysiological mechanisms, there is concern that significant differences may exist in some etiopathogenic factors. This study aims to compare the etiologies and risk factors of patients with anterior and posterior circulation strokes to ascertain if the operating mechanisms are any different and warrant different interventions.

Methods: A retrospective study compared the etiologies, risk factors, and stroke severity of 350 patients diagnosed with either anterior circulation infarcts (ACI) or posterior circulation infarcts (PCI) confirmed by magnetic resonance imaging. Stroke etiologies were classified according to the Trial of Organization 10172 in Acute Stroke Treatment (TOAST) criteria.

Results: The sample included 254 patients (72.6%) with ACI and 96 patients (27.4%) with PCI. Patients with PCI had a lower mean NIHSS score on admission (6.05 versus 8.70, p<0.001) and a lower mRS score at discharge (1.91 versus 2.48, p=0.004). The most frequent etiology for both types of strokes was large-artery occlusion, occurring in 77.1% of patients with PCI and 61.4% of those with ACI. PCI patients showed a significantly higher proportion of diabetes mellitus (80.2% versus 68.1%, p=0.025) and hypertension (82.2% versus 67.3%, p=0.006) as compared to patients with ACI. Other risk factors and etiologies were similar across both ACI and PCI.

Conclusions: Our study of South Indian patients showed that the most critical etiology for ACI and PCI was large artery atherosclerosis (LAA), which was relatively more frequent in patients with PCI. Patients with ACI have more severe strokes compared to PCI. Hypertension and diabetes were the more commonly encountered risk factors for PCI than for ACI. Our results imply that mechanisms of stroke for patients with both ACI and PCI are mostly similar, and treatment should address this correlation rather than focus on other differences. Stricter control of diabetes and hypertension may be warranted for patients with PCI, considering the more significant role attributed to these risk factors in this stroke category.

## Introduction

Approximately 80% of strokes are ischemic, with PCI accounting for about 20% of these cases [1]. However, there are significant geographical variations in the proportion of ACI and PCI in the ischemic stroke burden. Many clinicians regard PCI as different from ACI in risk factors, etiology, clinical manifestations, and prognosis [2]. Many studies done in the past examining these differences have varied in their use of imaging modalities. False-negative imaging occurs more frequently in PCI than in ACI, particularly within the initial hours following a stroke. MRI, compared to CT, also demonstrates a superior diagnostic yield for PCI [3]. We did a retrospective study comparing the etiologies, risk factors, and stroke severity of 350 patients with a diagnosis of either ACI or PCI, confirmed by a standard MRI protocol.

## Materials and methods

Study design

Data on 350 patients with acute ischemic stroke who presented to a tertiary care center in south India during the last five years were collected from the Electronic Medical Records (EMR) and Picture Archiving and Communication System (PACS). All patients received a clinical diagnosis of ischemic stroke according to the World Health Organization criteria [4]. A repeat MR imaging was done after 72 hours in cases of MRI-negative stroke [3]. Patients without an MRI-verified ischemic lesion, those with multiple infarcts involving both anterior and posterior circulation, and those with watershed zone infarcts between the anterior and posterior circulation were excluded from the study. We analyzed the remaining patients and classified them as having infarctions involving only the posterior circulation or only the anterior circulation.

Data collection

Data was collected from the EMR and PACS. It included patient demographics (age and sex), time of stroke onset, stroke severity assessed by the National Institute of Health Stroke Scale (NIHSS), modified Rankin Scale (mRS), and risk factors (hypertension, diabetes mellitus, dyslipidemia, atrial fibrillation, stroke in the past, coronary artery disease, and chronic kidney disease). The MRI images (1.5-Tesla) were carefully analyzed independently by two investigators (J.J.V. and S.V.). In addition to the blood tests (hematology, biochemistry, and coagulation studies; lipid profile), 12-lead electrocardiography and echocardiography were done to ascertain the stroke etiology and risk factors in individual cases. In a few cases, other specific tests such as a thrombophilia workup, carotid artery ultrasound, transcranial Doppler, and ambulatory electrocardiography were done. The stroke subtype was classified according to the TOAST categories based on clinical features, radiological findings, and other diagnostic tests [5,6].

Statistical analysis

The Pearson chi-squared test compared etiological and other categorical variables between the PCI and ACI groups. The Mann-Whitney U test was used to compare the means of continuous variables. When appropriate, odds ratios (ORs) were calculated with 95% confidence intervals. Significance was assessed using 2-tailed p-values, with the threshold defined as p<0.05. All statistical analyses were performed using SPSS Inc. Released 2007. SPSS for Windows, Version 16.0. Chicago, SPSS Inc.

## Results

Baseline characteristics

**Figure 1:**
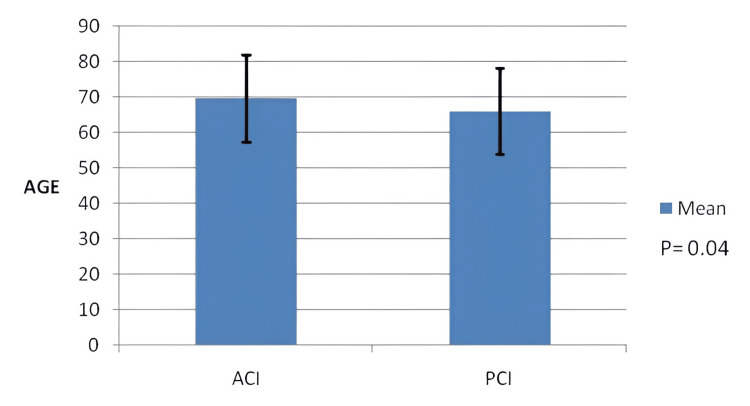
Mean age of subjects with standard deviation ACI: Anterior Circulation Infarction, PCI: Posterior Circulation Infarction

**Figure 2:**
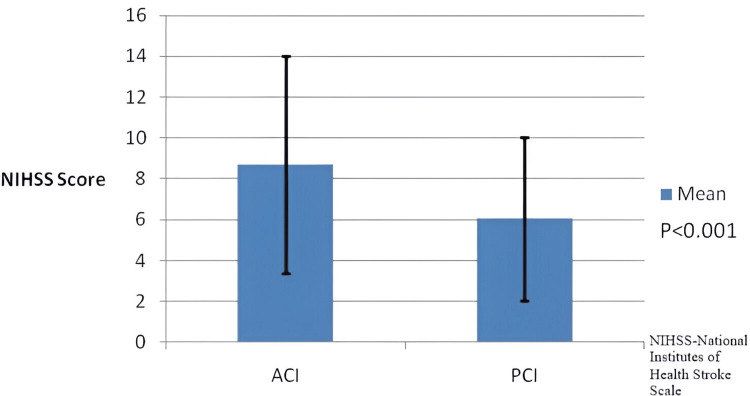
Mean NIHSS score of subjects with standard deviation ACI: Anterior Circulation Infarction, PCI: Posterior Circulation Infarction, NIHSS: National Institute of Health Stroke Scale

**Figure 3:**
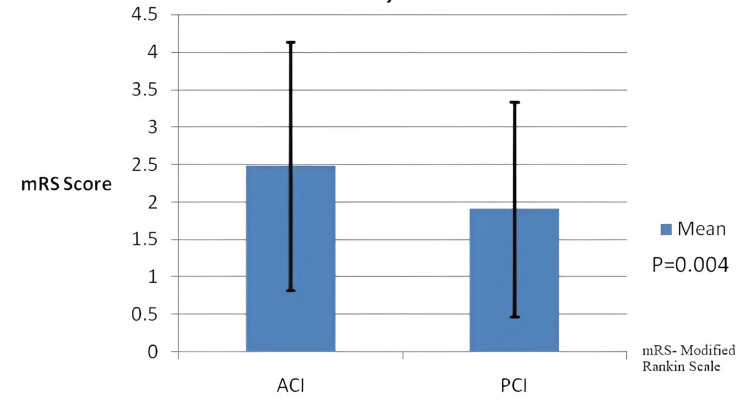
Mean mRS score of subjects with standard deviation ACI: Anterior Circulation Infarction, PCI: Posterior Circulation Infarction, mRS: modified Rankin Scale

A total of 350 patients (207 men, 59.1%) with a mean age of 68.67 ± 12.29 years were enrolled in this study (Figure 1). The majority of the ischemic strokes were ACI (72.6%), while 27.4% had PCI. The mean NIHSS score on admission was 6.14 ± 6.00. Compared to patients with ACI, PCI patients had a lower mean NIHSS score on admission (8.70 versus 6.05, p<0.001) and a lower mean mRS score at discharge (2.48 versus 1.91, p=0.004) (Figures 2, 3).

Risk factors

Both patient groups showed a similar distribution of risk factors, with relative frequencies in the order of hypertension (71.4%), diabetes mellitus (71.4%), dyslipidemia (32.9%), coronary artery disease (24.3%), previous ischemic stroke (16.3%), atrial fibrillation (12.6%), and chronic kidney disease (12.3%). However, PCI patients, as compared to patients with ACI, showed a significantly higher proportion of diabetes mellitus (80.2% versus 68.1%, p=0.025) and hypertension (82.2% versus 67.3%, p=0.006) (Table 1).

**Table 1:** Risk Factors of ACI and PCI *- shows statistical significance Data are in n (%) ACI: Anterior Circulation Infarction, PCI: Posterior Circulation Infarction, CAD: Coronary Artery Disease, CKD: Chronic Kidney Disease

Risk factors, n (%)	Total (n=350)	ACI (n=254)	PCI (n=96)	CHI-SQUARE	p-value
Hypertension	250 (71.4%)	171 (67.3%)	79 (82.2%)	7.649	0.006*
Diabetes mellitus	250 (71.4%)	173 (68.1%)	77 (80.2%)	4.997	0.025*
Dyslipidemia	115 (32.9%)	76 (29.9%)	39 (40.6%)	3.618	0.057
Atrial fibrillation	44 (12.6%)	35 (13.7%)	9 (9.3%)	1.23	0.267
Stroke in the past	57 (16.3%)	40 (15.7%)	17 (17.7%)	0.196	0.658
CAD	85 (24.3%)	58 (22.8%)	27 (28.1%)	1.06	0.303
CKD	43 (12.3%)	30 (11.8%)	13 (13.5%)	0.194	0.66

Stroke etiology

The frequency of stroke as per the TOAST categorization of patients for both PCI and ACI is shown in Table 3. Across both ACI and PCI groups, the most frequent etiology was LAA (65.7%), followed by small-artery occlusion (21.1%) and cardioembolism (11.1%). Other determined etiologies comprised only 0.6% of patients, while undetermined etiologies were noted in 0.7% of ACI and 3.1% of PCI. LAA was more frequent in PCI than ACI (77.1% versus 61.4%, p=0.010), while small artery occlusion had a significantly higher occurrence in the ACI group (25.2% vs. 10.4%, p=0.003). The two patient groups had no significant differences in other etiologic categories (Table 2).

**Table 2:** Etiology of ACI and PCI *- shows statistical significance Data are in n (%) ACI: Anterior Circulation Infarction, PCI: Posterior Circulation Infarction

Etiology	Total (n= 350)	ACI (n=254)	PCI (n=96)	CHI-SQUARE	p-value
Large-artery atherosclerosis	230 (65.7%)	156 (61.4%)	74 (77.1%)	6.587	0.010*
Small-artery occlusion	74 (21.1%)	64 (25.2%)	10 (10.4%)	9.128	0.003*
Cardioembolism	39 (11.1%)	31 (12.2%)	8 (8.3%)	1.055	0.304
Stroke of other determined etiology	2 (0.6%)	1 (0.3%)	1 (1.0%)	0.515	0.474
Stroke of undetermined etiology	5 (1.4%)	2 (0.7%)	3 (3.1%)	2.703	0.129

## Discussion

Infarcts involving the anterior and posterior circulation are generally thought to share the same pathophysiological mechanisms. However, there is concern that some etiopathogenic factors may differ between these groups, which may affect the treatment [7]. There is wide geographical variability among studies on stroke etiologies from various countries as per the TOAST classification. Our study also shows significant differences in stroke etiologies compared to neighboring countries. LAA was the most common etiology for both ACI and PCI in our study, similar to most other studies [8-11]. However, studies from China and Taiwan reported small vessel disease to be the most prevalent ischemic stroke subtype [12,13]. Small vessel disease was also the most pervasive stroke subtype in studies from Jordan (36%), Japan (54.1%), and Kuwait (69.8%) [14-16]. In a study from Indonesia, cardioembolism accounted for only 2.1%, while 9.8 % were of undetermined etiology, compared to 11.1% and 1.4%, respectively, in our study [8]. However, in another study from Pakistan, cardioembolism accounted for as much as 40% of their cases and was the largest stroke subtype [17]. The stroke etiologies also differed between anterior and posterior circulations in our study, with a higher contribution of LAA observed in patients with PCI. Meanwhile, we noted a relatively larger share of small artery occlusion (25.2% vs. 10.4%) and cardioembolism (12.2% vs. 8.3%) in ACI compared to PCI. Unlike our results, small artery disease was significantly more frequent in PCI (16.1%) than ACI (7.4%, p=0.01) in a study from Switzerland [18].

Among the risk factors for stroke, diabetes, and hypertension were observed with a higher frequency in PCI in our study. The association of PCI with diabetes was also noted in other observational studies [19-21]. A study in China also found that hypertension was more associated with PCI than ACI, although the reason for this couldn’t be established [22]. While some researchers have noted that diabetes has a closer link to small vessel disease, others have pointed out a correlation to LAA [23]. This indicates that the stroke etiology, as per the TOAST classification alone, cannot explain the closer association of diabetes with PCI.

In our study, ACI patients were older and more likely to suffer disability and death, as evidenced by the higher mRS score at discharge. There was also a significant difference in NIHSS scores between ACI and PCI, potentially because the scoring system gives more weight to ACI deficits and does not account for disabling deficits specific to PCI, such as gait abnormalities and vertigo [24].

Despite the broad similarities in the etiology and risk factors for ACI and PCI, there may be significant differences in some aspects of stroke mechanisms, particularly concerning large artery occlusion. Posterior circulation intracranial atherosclerosis was reportedly associated more with metabolic derangement and local branch occlusion than anterior circulation, where artery-to-artery embolism was the predominant mechanism. This influences the outcome of their management concerning endovascular thrombectomy [25].

Our study on South Indian patients suggests common stroke etiologies and risk factors for patients with both ACI and PCI, as in most other studies, with only subtle differences. Hence, it is reasonable that patients with PCI and ACI should be treated in the same way rather than differently based on their anatomical differences. However, with regard to secondary stroke prevention in patients with TIA or infarction involving the posterior circulation, our results indicate that an aggressive strategy for the control of diabetes and hypertension, as well as preventive measures for LAA, may be warranted. A major limitation of our study is the inherent drawbacks associated with the retrospective study design. Some risk factors, like smoking, family history of stroke, and BMI, couldn’t be included because of a lack of uniform data. We also did not include a control group without strokes, which could have yielded more robust results.

## Conclusions

Our study of South Indian patients with PCI and ACI showed similar etiologies and risk factors as those from most other geographical locations. Patients with PCI had a lower mean age of occurrence and lower mean NIHSS and mRS scores. LAA, diabetes, and hypertension were commonly noted in patients with PCI as compared to those with ACI. While the etiopathogenic differences observed between ACI and PCI in our study do not justify risk factor stratification or different treatment modalities, they may have a role in secondary stroke prevention for stroke or TIA involving the posterior circulation.

## References

[1] Savitz SI, Caplan LR (2005). Vertebrobasilar disease. N Engl J Med.

[2] Caplan L (2000). Posterior circulation ischemia: then, now, and tomorrow. The Thomas Willis Lecture-2000. Stroke.

[3] Castaneda CL, Rhee JA, Woo JH, Lerario MP (2020). False-negative initial magnetic resonance imaging in acute posterior circulation stroke: a case report describing locked-in syndrome. Cureus.

[4] Aho K, Harmsen P, Hatano S, Marquardsen J, Smirnov VE, Strasser T (1980). Cerebrovascular disease in the community: results of a WHO Collaborative Study. Bull World Health Organ.

[5] Adams HP Jr, Bendixen BH, Kappelle LJ, Biller J, Love BB, Gordon DL, Marsh EE 3rd (1993). Classification of subtype of acute ischemic stroke. Definitions for use in a multicenter clinical trial. TOAST. Trial of Org 10172 in Acute Stroke Treatment. Stroke.

[6] Chung JW, Park SH, Kim N (2014). Trial of ORG 10172 in Acute Stroke Treatment (TOAST) classification and vascular territory of ischemic stroke lesions diagnosed by diffusion-weighted imaging. J Am Heart Assoc.

[7] Kim JS, Nah HW, Park SM (2012). Risk factors and stroke mechanisms in atherosclerotic stroke: intracranial compared with extracranial and anterior compared with posterior circulation disease. Stroke.

[8] Harris S, Sungkar S, Rasyid A, Kurniawan M, Mesiano T, Hidayat R (2018). TOAST subtypes of ischemic stroke and its risk factors: a hospital-based study at Cipto Mangunkusumo Hospital, Indonesia. Stroke Res Treat.

[9] Bejot Y, Caillier M, Ben Salem D (2008). Ischaemic stroke subtypes and associated risk factors: a French population based study. J Neurol Neurosurg Psychiatry.

[10] Porcello Marrone LC, Diogo LP, de Oliveira FM (2013). Risk factors among stroke subtypes in Brazil. J Stroke Cerebrovasc Dis.

[11] Deleu D, Inshasi J, Akhtar N (2011). Risk factors, management and outcome of subtypes of ischemic stroke: a stroke registry from the Arabian Gulf. J Neurol Sci.

[12] Zeng Q, Tao W, Lei C, Dong W, Liu M (2015). Etiology and risk factors of posterior circulation infarction compared with anterior circulation infarction. J Stroke Cerebrovasc Dis.

[13] Hsieh FI, Chiou HY (2014). Stroke: morbidity, risk factors, and care in Taiwan. J Stroke.

[14] Bahou Y, Hamid H, Hadidi A (2004). Ischaemic stroke in Jordan: a 2-year hospital-based study of subtypes and risk factors. East Mediterr Health J.

[15] Turin TC, Kita Y, Rumana N (2010). Ischemic stroke subtypes in a Japanese population: Takashima Stroke Registry, 1988-2004. Stroke.

[16] Al-Hashel JY, Al-Sabah AA, Ahmed SF (2016). Risk factors, subtypes, and outcome of ischemic stroke in Kuwait: a national study. J Stroke Cerebrovasc Dis.

[17] Zafar F, Tariq W, Shoaib RF, Shah A, Siddique M, Zaki A, Assad S (2018). Frequency of ischemic stroke subtypes based on toast classification at a tertiary care center in Pakistan. Asian J Neurosurg.

[18] De Marchis GM, Kohler A, Renz N (2011). Posterior versus anterior circulation strokes: comparison of clinical, radiological and outcome characteristics. J Neurol Neurosurg Psychiatry.

[19] Karapanayiotides T, Piechowski-Jozwiak B, van Melle G, Bogousslavsky J, Devuyst G (2004). Stroke patterns, etiology, and prognosis in patients with diabetes mellitus. Neurology.

[20] Arboix A, Rivas A, García-Eroles L, de Marcos L, Massons J, Oliveres M (2005). Cerebral infarction in diabetes: clinical pattern, stroke subtypes, and predictors of in-hospital mortality. BMC Neurol.

[21] Subramanian G, Silva J, Silver FL (2009). Risk factors for posterior compared to anterior ischemic stroke: an observational study of the Registry of the Canadian Stroke Network. Neuroepidemiology.

[22] Li Y, Cai Y, Zhao M, Sun J (2017). Risk factors between intracranial-extracranial atherosclerosis and anterior-posterior circulation stroke in ischaemic stroke. Neurol Res.

[23] Tuttolomondo A, Pinto A, Salemi G (2008). Diabetic and non-diabetic subjects with ischemic stroke: differences, subtype distribution and outcome. Nutr Metab Cardiovasc Dis.

[24] Kim JT, Park MS, Choi KH (2017). Clinical outcomes of posterior versus anterior circulation infarction with low National Institutes of Health Stroke Scale Scores. Stroke.

[25] Mbroh J, Poli K, Tünnerhoff J (2021). Comparison of risk factors, safety, and efficacy outcomes of mechanical thrombectomy in posterior vs. anterior circulation large vessel occlusion. Front Neurol.

